# Supplementary material for: Single-cell sequencing deconvolutes cellular responses to exercise in human skeletal muscle
Source: Commun Biol. 2022 Oct 22;5:1121. doi: 10.1038/s42003-022-04088-z (PMC9588010; doi:10.1038/s42003-022-04088-z)
Supplement: Supplementary file 2 — Supplementary Information [file 42003_2022_4088_MOESM2_ESM.pdf]

Supplementary Materials:
Supplementary references 1 and 2

Supplementary Figure 1: Comparison of pre- and post-single bout of exercise across the UMAP.
Supplementary Figure 2: Marker gene expression across all subpopulations.

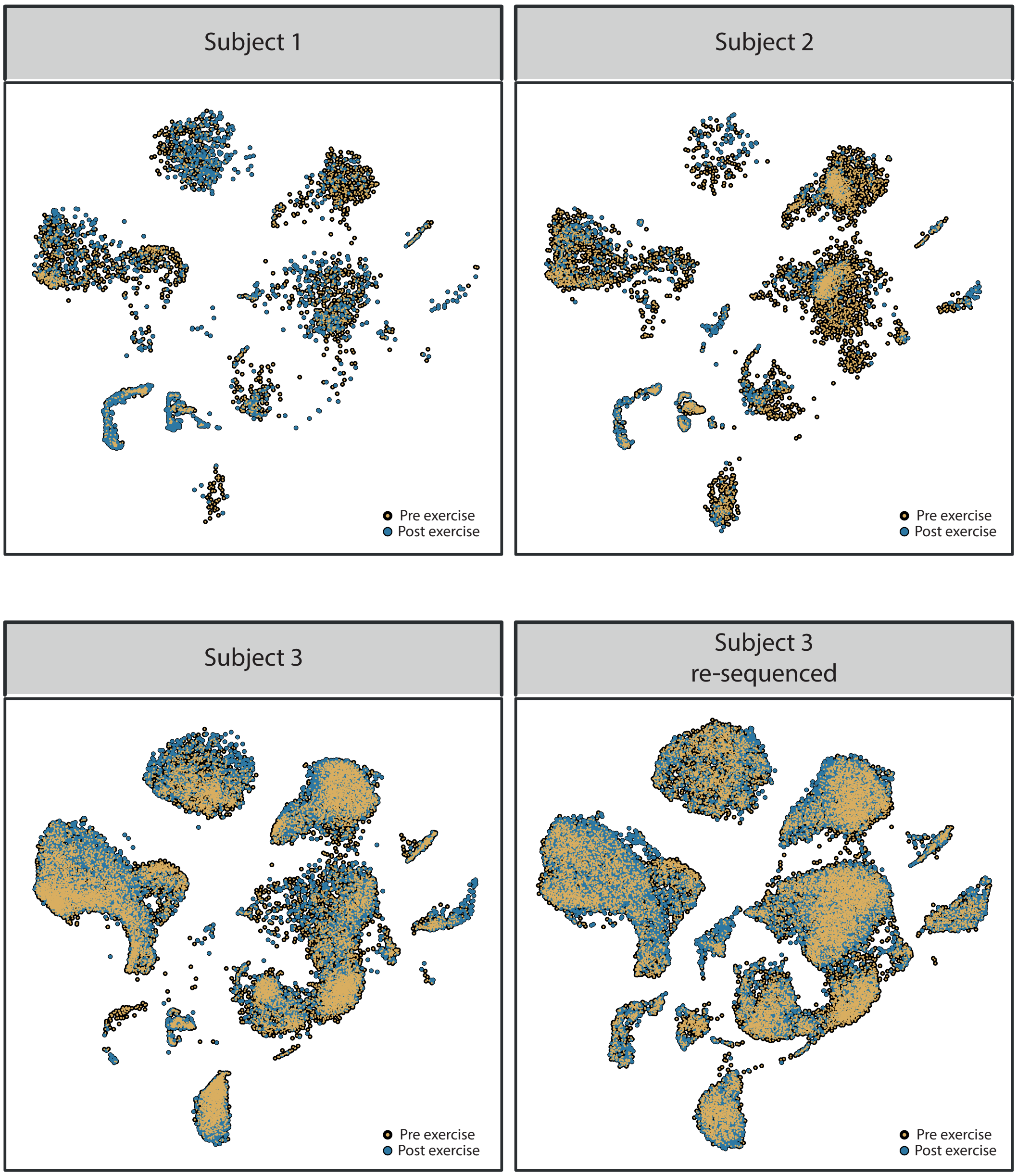

Supplementary Figure 1. Comparison of pre- and post single bout of exercise across the UMAP. Presented are the UMAP visualizations of each sample comparing the local alignment pre- and post-exercise bout. Although the subjects were characterised by differences in absolute numbers of cells, all identified cell types contained cells from all samples, irrespective of subject or exercise-bout. This highlights the reproducibility of the identified cell types.

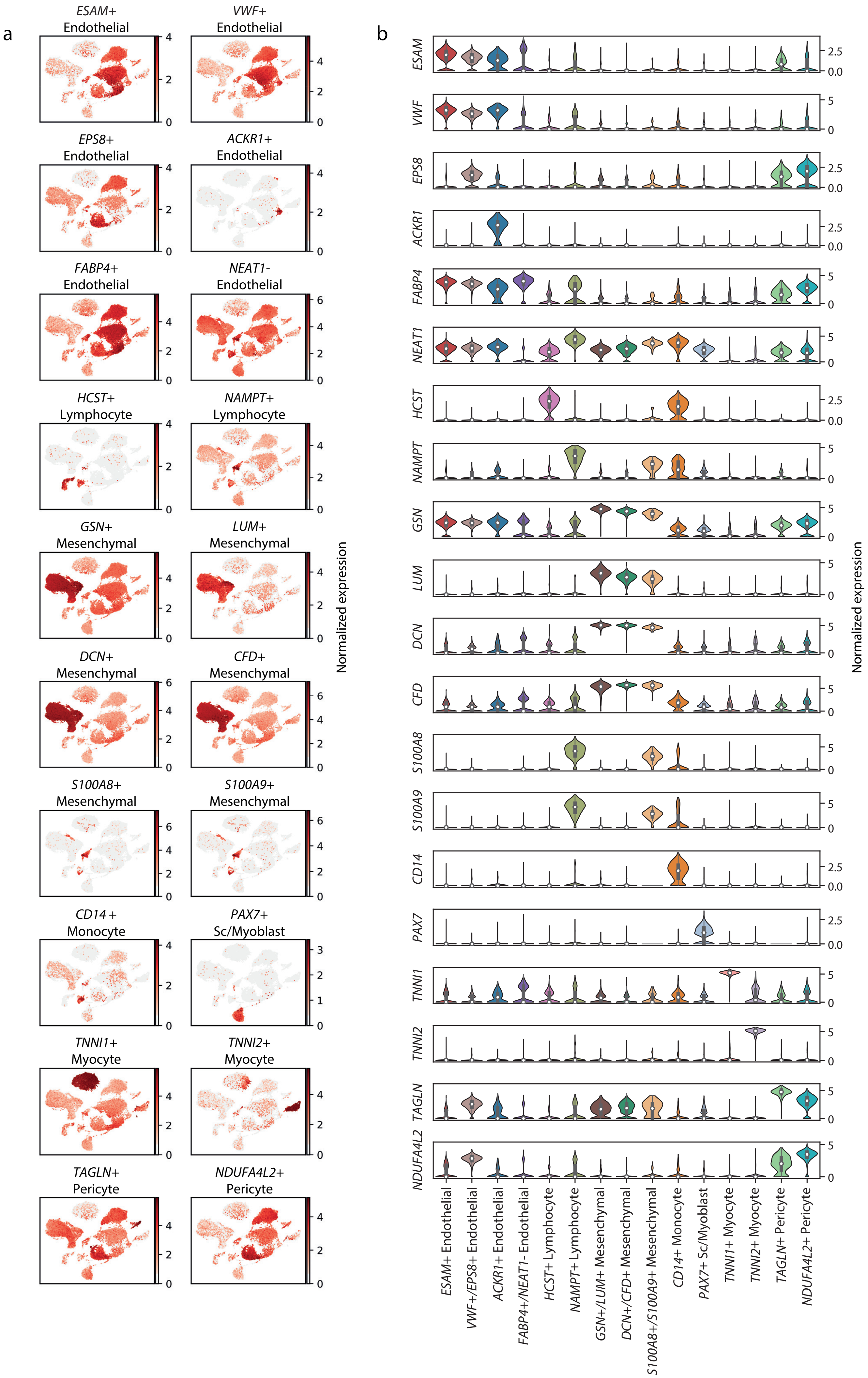

a
ESAM+ Endothelial
VWF+ Endothelial
EPS8+ Endothelial
ACKR1+ Endothelial
FABP4+ Endothelial
NEAT1- Endothelial
HCST+ Lymphocyte
NAMPT+ Lymphocyte
GSN+ Mesenchymal
LUM+ Mesenchymal
DCN+ Mesenchymal
CFD+ Mesenchymal
S100A8+ Mesenchymal
S100A9+ Mesenchymal
CD14 + Monocyte
PAX7+ Sc/Myoblast
TNNI1+ Myocyte
TNNI2+ Myocyte
TAGLN+ Pericyte
NDUFA4L2+ Pericyte
Normalized expression
b
ESAM
VWF
EPS8
ACKR1
FABP4
NEAT1
HCST
NAMPT
GSN
LUM
DCN
CFD
S100A8
S100A9
CD14
PAX7
TNNI1
TNNI2
TAGLN
NDUFA4L2
Normalized expression
ESAM+ Endothelial
VWF+/EPS8+ Endothelial
ACKR1+ Endothelial
FABP4+/NEAT1- Endothelial
HCST+ Lymphocyte
NAMPT+ Lymphocyte
GSN+/LUM+ Mesenchymal
DCN+/CFD+ Mesenchymal
S100A8+/S100A9+ Mesenchymal
CD14+ Monocyte
PAX7+ Sc/Myoblast
TNNI1+ Myocyte
TNNI2+ Myocyte
TAGLN+ Pericyte
NDUFA4L2+ Pericyte

Supplementary Figure 2. Marker gene expression across all subpopulations. (a) UMAPs highlighting marker genes of a given cell type. The colormap legend highlights the normalized expression levels used to color the UMAP. **(b)** Normalized expression of given marker genes against a given cell subpopulation. The data in boxplots nested within violin plots are expressed as median, interquartile range, minimum and maximum values. **(a-b)** Correspond to each other. The normalized expression refers to log(1+x) if not stated otherwise. Sc, satellite cells.
