# Supplementary material for: Single-cell sequencing deconvolutes cellular responses to exercise in human skeletal muscle
Source: Commun Biol. 2022 Oct 22;5:1121. doi: 10.1038/s42003-022-04088-z (PMC9588010; doi:10.1038/s42003-022-04088-z)
Supplement: Supplementary file 3 — Description of Additional Supplementary Files [file 42003_2022_4088_MOESM3_ESM.docx]

**Supplementary Data 1.**

**Composition tables.** Represents calculated cell type composition for individual samples

**Supplementary Data 2.**

**Enrichment analysis.** Gene ontology enrichment analysis considering significantly up-regulated genes (*fdr*<0.05) for annotated cell types at baseline.

**Supplementary Data 3.**

**Differential expression.** Represents differentially expressed genes (biomarkers) for individual cell types at baseline.

**Supplementary Data 4.**

**Differential expression.** Represents differentially expressed genes (biomarkers) for cell type subpopulations at baseline.

**Supplementary Data 5.**

**Differential expression.** Represents differentially expressed genes for individual cell types after single bout of exercise.

**Supplementary Data 6.**

**Enrichment analysis.** Gene ontology enrichment analysis considering significantly up-regulated genes (*fdr*<0.05) for annotated subpopulations after single bout of exercise.

**Supplementary Data 7.**

**Differential expression.** Represents differentially expressed genes for annotated cell type subpopulations after single bout of exercise.

**Supplementary Data 8.**

**Differential expression.** Represents the list of significantly upregulated genes post-exercise across all different cell types, and corresponding logFC.

**Supplementary Data 9.**

**Enrichment analysis – myogenic differentiation.** Gene ontology enrichment analysis considering up and down regulated genes (*fdr*<0.05) for annotated myogenic subpopulations after single bout of exercise.

**Supplementary Data 10.**

**The source data underlying figures.** Individual single-cell gene expression levels of marker genes, exercise differentially expressed genes across all cell populations including RT-PCR and microarray expression data from primary myoblasts and C2C12 cells respectively. It also contains pseudotime-data for myogenic cells.
